# Genome sequence of N_2_-fixer *Stutzerimonas stutzeri* strain MBI-RS3

**DOI:** 10.1128/mra.01192-24

**Published:** 2025-09-17

**Authors:** Patricia Dörr de Quadros, Julie E. Hernandez-Salmeron, Michael D.J. Lynch, Jiujung Cheng, Trevor C. Charles

**Affiliations:** 1 Department of Biology, University of Waterloo 8430, Waterloo, Canada; 2 Earth Microbial Canada Inc., Waterloo, Ontario, Canada; 3 Metagenom Bio Life Science Inc., Waterloo, Canada; The University of Arizona, Tucson, Arizona, USA

**Keywords:** *Stutzerimonas*, plant-microbe interaction, biological N fixation, plant growth promotion

## Abstract

We announce the complete genome sequence of *Stutzerimonas stutzeri* strain MBI-RS3, a rifampicin-resistant derivative found in a mixed bacterial culture at the University of Waterloo. This strain enhances plant growth via nitrogen fixation, phosphate solubilization, and the production of trehalose and phytohormones.

## ANNOUNCEMENT

*Stutzerimonas* is a recently defined genus within the *Pseudomonadaceae*, known for its adaptability and metabolic range (1, 2). Strain MBI-RS3 was discovered as a contaminant in an *Azospirillum* sp. stock tube from a −80 freezer, originally isolated from agricultural soil at the University of Waterloo (archival reference unavailable).

Rifampicin resistance was detected when colonies grew on tryptic soy agar (TSA) supplemented with 50 µg/mL rifampicin, indicating spontaneous development in storage rather than selection in the laboratory. Cells from a single colony streaked on TSA were inoculated into tryptic soy broth and incubated at 30°C, 160 rpm for 30 h. An aliquot was re-streaked on TSA to confirm purity, and a single colony was used for DNA extraction. DNA was extracted using the Sox Soil DNA Extraction Kit (Metagenom Bio) and quantified with a Qubit dsDNA assay (Thermo Fisher). Illumina libraries were prepared with the DNA Prep Kit (formerly Nextera Flex), sequenced on a MiSeq platform (2×251 bp), yielding 10 million paired-end reads (5.0 Gbp; GC 57.7%; N50: 251 bp) (SRA: SRR32266762). Nanopore libraries were prepared using the Rapid Barcoding Kit 96 (SQK-RBK110.96, ONT) and sequenced on a MinION with an R9.4.1 flow cell, yielding 4,000 reads (33.1 Mbp; avg. 8,270 bp; N50 ~9–10 kb; GC 64%) (SRA: SRR32266763). Guppy v6.1.5 (3) was used for base calling in high-accuracy mode; adapter trimming was done with Porechop (4).

Hybrid genome assembly was performed with Unicycler v0.5.0 (5), integrating Illumina and Nanopore reads. Short reads were first assembled with SPAdes v3.15.4 (6), and long reads incorporated via Miniasm (7). The resulting genome included two circular replicons: a chromosome (4,712,513 bp) and a plasmid (156,519 bp), with 717× coverage. Circularity was confirmed by graph visualization in Bandage v0.8.1 (8).

**Fig 1:**
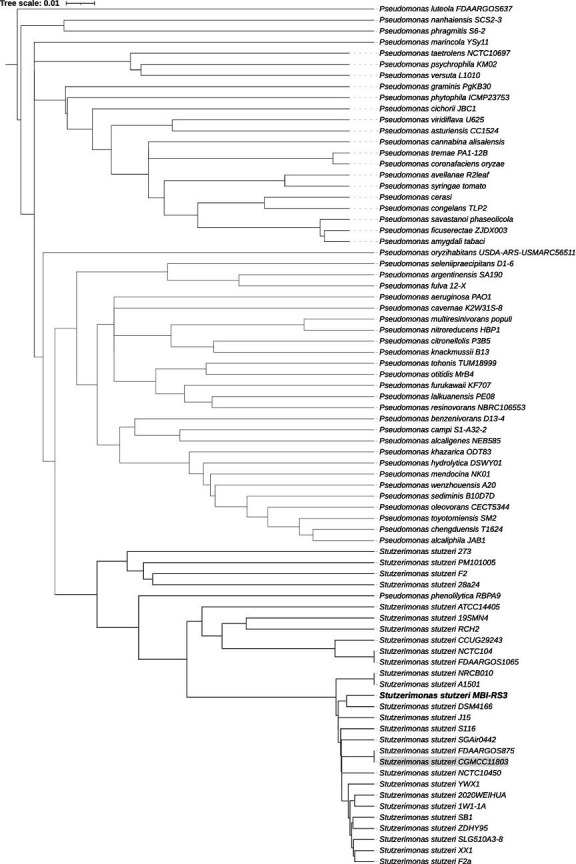
Fragment of hierarchical clustering based on the distances obtained with FastANI including species of *Pseudomonas* and *Stutzerimonas.*

Annotation with PGAP v6.8 (9) identified 4,616 genes, including 4,484 protein-coding sequences. CheckM v1.2.3 (10) reported 99.47% completeness and 0.2% contamination, ranking the genome in the top quartile of *S. stutzeri* RefSeq entries. FastANI v1.32 (11) showed 97.5% identity with the *S. stutzeri* type strain CGMCC11803, confirming species identity. Comparative analysis against 170 genomes (142 *Pseudomonas*, 28 *S*. *stutzeri*) clustered MBI-RS3 within the *S. stutzeri* clade, distinct from *Pseudomonas* (Fig. 1), based on hierarchical clustering of FastANI distances, visualized in iTOL (12).

The genome encodes key nitrogen fixation genes (*nifH, nifD, and nifK*) (Table 1), along with genes for rhizosphere colonization, chemotaxis, and plant-growth promotion. Phenotypic profiling using BIOLOG GenIII plates revealed the strain’s ability to metabolize a wide variety of sugars, amino acids, and organic acids, including dextrin, D-glucose, D-galactose, mannitol, gluconic acid, and L-malic acid, and tolerated 8% NaCl. This genome provides a valuable resource to investigate diazotrophy and plant-microbe interactions in this metabolically versatile species.

**TABLE 1:** Nitrogen fixation genes in *S. stutzeri* MBI-RS3

Gene	Start - End	AA length	Strand
nifM	3,363,266 - 3,364,144	879	←^*a*^
nifW	3,364,624 - 3,364,971	348	←
nifV	3,366,260 - 3,367,405	1,146	←
nifS	3,367,446 -3,368,654	1,209	←
nifU	3,368,656 - 3,369,606	951	←
nifN	3,378,179 - 3,379,555	1,377	←
nifE	3,379,566 - 3,380,987	1,422	←
nifT	3,383,145 - 3,383,366	222	←
nifK	3,383,458 - 3,385,029	1,572	←
nifD	3,385,132 - 3,386,613	1,482	←
nifH	3,386,725 - 3,387,606	882	←
nifL	3,396,823 - 3,398,373	1,551	→
nifA	3,398,387 - 3,399,952	1,566	→
nifB	3,404,934 - 3,406,448	1,515	→

^*a*^ The symbols → and ← are used to represent the forward (5′→3′) and reverse (3′→5′) directions of DNA strands, respectively.

## Data Availability

The genome is available in GenBank (BioProject: PRJNA1165279; BioSample: SAMN43929189; Assembly: ASM4269169v1; RefSeq: GCF_042691695.1). The raw sequencing reads are deposited in the NCBI Sequence Read Archive under accession numbers SRR32266763 (ONT) and SRR32266762 (Illumina).
